# Obstetric Synopsis

**Published:** 1888-04

**Authors:** 


**Obstetric Synopsis.** By JOHN S. STEWART, M. D., Demonstrator of Obstetrics, and Chief Assistant in the Gynecological Clinic of the Medico-Chirurgical College of Philadelphia; illustrated. Philadelphia: F. A. Davis, publisher. 1888. Pp. 200.

This little work belongs to the "Physicians' and Students' Ready Reference Series." It is well written, excellently illustrated, and fully up to date in every respect. Here we find all the essentials of obstetrics in a nutshell; anatomy, embryology, physiology, pregnancy, labor, puerperal state and obstetric operations, all being carefully and accurately described. In an appendix is given the obstetrical nomenclature adopted by the Obstetric Section of the Ninth International Medical Congress, of 1887.

